# Severe fibromyalgia alleviated by the unique muscle relaxation method of applying low force: A case report

**DOI:** 10.1097/MD.0000000000037929

**Published:** 2024-04-19

**Authors:** Haruka Amitani, Ryusei Nishi, Takamasa Fukumoto, Kazumasa Hamada, Ryuichi Kato, Takako Yamamoto, Yuuki Fuku, Kenichiro Sagiyama, Akihiro Asakawa

**Affiliations:** a Department of Psychosomatic Internal Medicine, Kagoshima University Graduate School of Medical and Dental Sciences, Sakuragaoka, Kagoshima, Japan.

**Keywords:** case report, fibromyalgia, muscle relaxation method

## Abstract

**Rationale:**

Fibromyalgia (FM) is characterized by idiopathic persistent chronic pain in the ligaments or musculoskeletal system, and more than half of the patients with FM might have migraine headaches. Direct musculoskeletal intervention could be a non-pharmacological management to relieve symptoms. However, patients with severe FM often have intense pain from only a soft touch, thereby rendering musculoskeletal intervention challenging.

**Patient concerns:**

A 47-year-old man had progressing intense pain, and this affected his everyday life. There were no abnormal physical findings on laboratory examination such as levels of complement, antinuclear antibodies, and C-reactive protein, which were within normal limits. Magnetic resonance imaging did not indicate abnormalities.

**Diagnoses, interventions, and outcomes:**

The patient satisfied the American College of Rheumatology criteria. Finally, we made a final diagnosis of fibromyalgia. The therapeutic intervention of *Kanshoho*, the unique muscle relaxation technique with low force, relieved his pain.

**Lessons:**

If *Kanshoho* is carefully applied in a state of hospitalization under surveillance by an experienced physician, it could be a promising muscle relaxation method. Relaxing the trapezius muscle and reducing its intramuscular pressure might be key in treating patients with severe FM. However, it needs elucidation of its mechanism.

## 1. Introduction

Fibromyalgia (FM) is characterized by idiopathic persistent chronic pain in the ligaments or musculoskeletal system.^[1]^ Patients often exhibit aberrant pain perception, such as hyperalgesia (patients perceive pain more intensely than healthy individuals) or allodynia (unresponsive stimuli such as touch or temperature change are perceived as pain), suggesting that FM pathophysiology may be caused neurobiologically.^[2]^ However, symptoms are not always limited to pain, and most patients have comorbidities such as cognitive impairment, sleep disturbance (unrefreshed wakening), or fatigue.^[3]^ Additionally, an electronic survey showed that more than half of the respondents with FM suffers from migraine headache.^[4]^

Moreover, FM often overlaps with myalgic encephalomyelitis/chronic fatigue syndrome (ME/CFS). ME/CFS is diagnosed when patients have suffered from idiopathic fatigue consecutively for 6 months.^[5]^ Although the overlap has been discussed for a long time, a recent systematic review indicates that almost half of the reported cases overlapped FM and ME/CFS.^[6]^

Presently, FM does not have radical treatments. However, various treatments are suggested for relieving symptoms. For non-pharmacological treatments, only aerobic and strengthening exercise is strongly supported for patients with FM, by the European League Against Rheumatism, and other non-pharmacological treatments, such as mindfulness, acupuncture, hydrotherapy, and cognitive behavioral therapy, are concluded to have only weak evidence to support their practical use.^[7]^

Direct musculoskeletal intervention could be a non-pharmacological management to relieve symptoms. Recently, muscle relaxation therapy, one of the non-pharmacological forms of management, has been endorsed in a randomized controlled pilot trial for patients with FM.^[8]^ Additionally, a meta-analysis suggests the effectiveness of massage therapy.^[9]^ However, it should be noted that even if exercise or massage is effective, patients find difficulty in receiving treatments because of allodynia or hyperalgesia. Patients with severe FM often have intense pain from only a soft touch, rendering musculoskeletal intervention challenging.

Massage therapy or exercise was not applied to the patient reported here. However, his symptoms were considerably relieved by muscle relaxation therapy with low force.

## 2. Patient information and clinical findings

A written consent was obtained from the patient for publication of this case report accompanying images and videos. A 47-year-old man developed a fever, cough, and sore throat after being in close contact with a patient diagnosed positive for COVID-19. However, the result of the polymerase chain reaction test was negative. A week later, he suddenly experienced dyspnea as well as chest and back pain; he was immediately rushed to an emergency room. Nonetheless, no abnormalities were found in physiological and laboratory examinations. The fever of unknown origin, ranging from 37 °C to 38 °C continued for months. Additionally, he experienced malaise and pain in his right arm and elbow joints.

Upon examination, the rheumatologists found no abnormal physical findings. Levels of complement, antinuclear antibodies, and C-reactive protein were within normal limits, leading the rheumatologists to refer the patient to our department. Considering his desire for pharmacological therapy before the final diagnosis, we postponed further investigations. Despite pharmacological treatments, including eperisone, pregabalin, and tramadol, the pain persisted and spread to the entire body after 6 months. The patient requested further investigations, and the neurologists made no indication of abnormalities from examinations, including magnetic resonance imaging. Based on these findings, we made a definitive diagnosis of ME/CFS. The pain was intensified by the month, and he was not able to live a normal life. Most of the time, he laid on his bedroom. Ultimately, he found it difficult to cope with his pain at home, and this led him to be admitted to our department.

## 3. Diagnostic assessment

**Figure 1.**
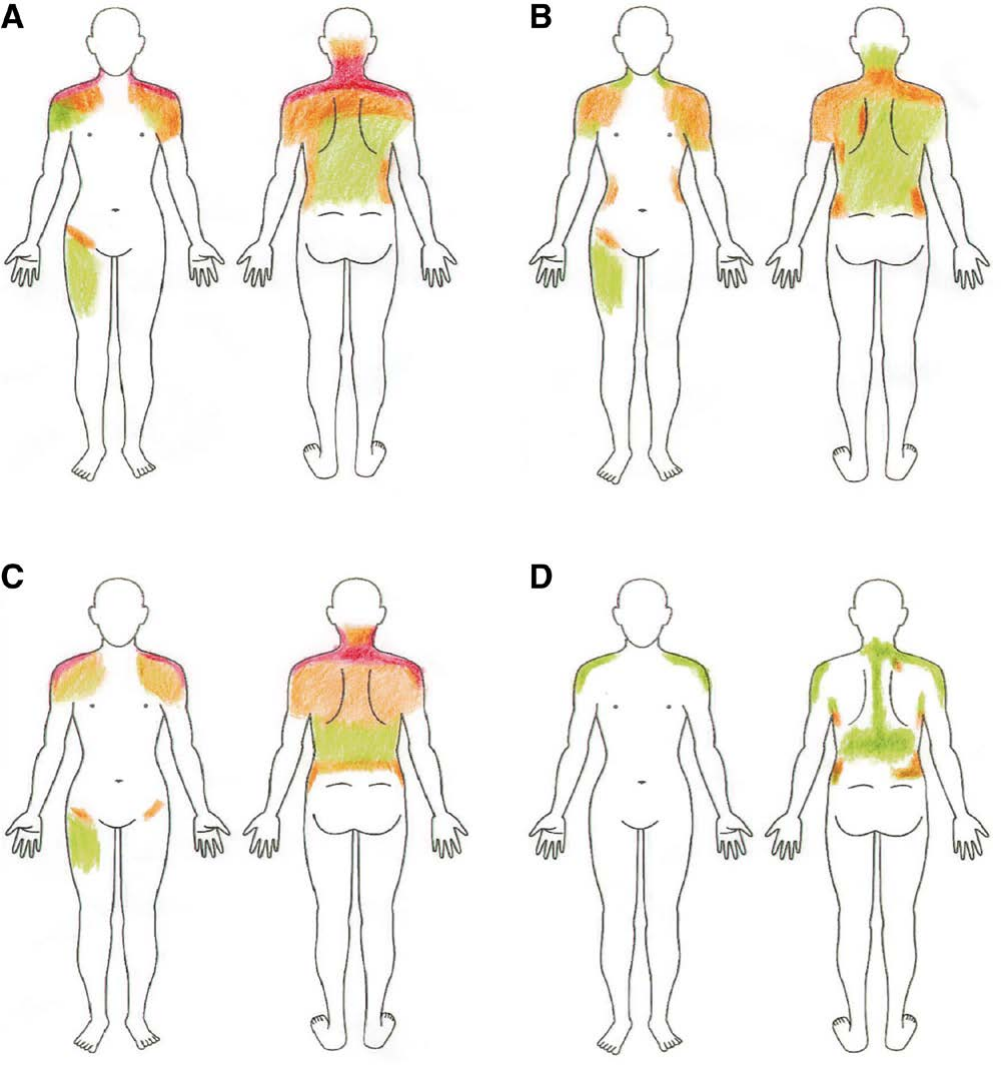
Pain intensity reported by the patient. The patient reported his pain using a color scale: red (intense), orange (moderate), and green (slight). (A) Day 1, (B) Day 7, (C) Day 12, and (D) Day 20.

Under the diagnosis of ME/CFS, his malaise and pain were considered idiopathic. Furthermore, applying a force of 100 g was enough for him to feel as if he was “being stabbed by a bed of nails,” suggesting allodynia. The pain was present in the right and left upper, axial, and right lower regions, suggesting generalized pain. The widespread pain index was 12 (Fig. 1A; the pain was absent on the bilateral jaw, chest, abdomen, and left lower region). The symptom severity score was 9 (severe fatigue and unrefreshed wakening, mild cognitive symptoms, headaches, and depression). Therefore, he satisfied the American College of Rheumatology criteria.^[3]^ Finally, we made a final diagnosis of fibromyalgia comorbid with ME/CFS.

## 4. Therapeutic intervention

### 4.1. Explanation of the unique muscle relaxation method of *Kanshoho*

Conventional muscle relaxation or massage therapy was impossible because of the allodynia. However, with a force of 100 g, we found that he could endure the pain at the superior nuchal line. Therefore, we performed the unique muscle relaxation method of *Kanshoho.*^[10]^ Although the mechanism needs elucidation, *Kanshoho* induces relaxation in skeletal muscle within an arbitrary area of approximately 1.0 cm^2^, when a force between 100 g and 500 g is applied, coupled with expansion and contraction lasting for approximately 2 seconds each. An example performance is available in Video File S1, Supplemental Digital Content, http://links.lww.com/MD/M254. Notably, each attempt can only relax within 1.0 cm^2^ of skeletal muscle. Therefore, to relax a targeted muscle, *Kanshoho* is performed by slightly changing the position where force is applied.

### 4.2. Therapeutic intervention with *Kanshoho*

On day 1, we performed *Kanshoho*, along the superior nuchal line for 30 minutes at a force of 100 g. Then, we extended the region to include the posterior neck on days 4 and 5, although the pain persisted for the first week. On day 7, he reported that his shoulder pain was relieved. He endured pain at the posterior neck and shoulder, with a force of 200 g. Therefore, we performed *Kanshoho* at this force on days 7, 8, and 10. On day 11, his pain was relieved; however, on day 12, he reported intense pain in his entire body (visual analog scale = 98 mm). Subsequently, we gradually and carefully applied the force from 100 g to 300 g and performed *Kanshoho* longer (60 minutes) than usual (30 minutes) to aim for deeper relaxation, until his pain was relieved. On days 13, 14, 17, and 18, he was again able to receive a *Kanshoho* of 300 g and reported relief from his pain. The region receiving *Kanshoho* gradually extended from the superior nuchal line (on day 1) to the Th7 spinous process, including the side of the neck and the superior part of the shoulders (on day 19). On day 19, he successfully received a *Kanshoho* of 400 g. Finally, on day 20, his pain was considerably relieved, as evidenced by the visual analog scale (Table 1).

**Table 1:** Visual analog scale of the pain performed after *Kanshoho.*

Days	Neck (mm)			Shoulder (mm)	
	Left	Right	Posterior	Right	Left
1	78	79	82	91	81
8	54	54	54	55	54
11	56	67	67	67	70
12	98	98	98	98	98
13	58	63	51	84	54
14	31	46	35	67	47
16	35	49	34	58	44
18	36	51	38	53	43
19	41	50	41	48	43
20	3	10	3	17	5

## 5. Outcomes

In Figure 1, manikins indicate the intensity of pain reported by the patient. Although his neck and shoulder pain remitted in the middle course of treatment, his pain was finally relieved.

## 6. Discussion

Recently, an intramuscular force of the trapezius muscle was significantly higher in patients with FM, suggesting that the stiffness of the trapezius may trigger FM.^[11]^ Additionally, Simms et al assessed 75 tender points and indicated that pressure intolerance was not significantly different between controls and patients with FM above the nuchal line (*P* = .55).^[12]^ The superior nuchal line is one of the origin points of the trapezius. Therefore, if FM is caused by elevated intramuscular pressure in the trapezius muscle, then their findings on pressure tolerance above the nuchal line are reasonable.

Nevertheless, even if muscle relaxation could be effective, conventional muscle relaxation is impossible for patients with FM because of allodynia. However, the unique muscle relaxation of *Kanshoho* applies only low force. Therefore, the patient may not feel pain above the level of the nuchal line, suggesting that the nuchal line is an appropriate region to start *Kanshoho*.

Although *Kanshoho* could be a unique way of relaxing muscles with low force, its mechanism is unknown. However, *Kanshoho* might be similar to myofascial release, aiming to release muscles from the fascial restriction, which makes connective tissue stiff.^[13]^

There are limitations when applying *Kanshoho*, considering that its mechanism is unknown. In patients with severe FM, such as the current patient, it may be desirable to introduce *Kanshoho* in a state of hospitalization under surveillance by an experienced physician. We experienced the maximum aggravation on day 12 and successfully alleviated the pain with extensive and careful use of *Kanshoho* from empirical management. The aggravation may have resulted from low barometric pressure. In patients with FM, barometric pressure is widely recognized as associated with FM.^[14,15]^ The patient’s complaint of increasing pain from day 11 is consistent with the onset of rainfall.

Reflecting on our present report, *Kanshoho* might be a promising muscle relaxation method, and relaxing the trapezius muscle and reducing its intramuscular pressure might be key in treating patients with severe FM. However, it needs elucidation of its mechanism.

## Author contributions

**Conceptualization:** Haruka Amitani, Ryusei Nishi, Akihiro Asakawa.

**Data curation:** Haruka Amitani, Takamasa Fukumoto, Kazumasa Hamada, Ryuichi Kato, Takako Yamamoto, Yuuki Fuku, Kenichiro Sagiyama.

**Investigation:** Haruka Amitani, Ryusei Nishi.

**Methodology:** Haruka Amitani.

**Visualization:** Takamasa Fukumoto.

**Writing – original draft:** Ryusei Nishi.

**Writing – review & editing:** Haruka Amitani, Ryusei Nishi, Takamasa Fukumoto, Kazumasa Hamada, Ryuichi Kato, Takako Yamamoto, Yuuki Fuku, Kenichiro Sagiyama, Akihiro Asakawa.

## Supplementary Material


